# Correlation Between Condylar Shape and Malocclusion: CBCT Analysis

**DOI:** 10.3390/diagnostics15060768

**Published:** 2025-03-19

**Authors:** Neamat Hassan Abubakr, Tanya Al-Talib, Nastaran Bahar, Arshia Badani, Stanely Nelson, Jyoti Mago

**Affiliations:** 1 Department of Biomedical Sciences, School of Dental Medicine, University of Nevada, Las Vegas, NV 89106, USA; 2 Department of Clinical Sciences, School of Dental Medicine, University of Nevada, Las Vegas, NV 89106, USA; tanya.al-talib@unlv.edu (T.A.-T.); stanley.nelson@unlv.edu (S.N.); jyoti.mago@unlv.edu (J.M.); 3 School of Dental Medicine, University of Nevada, Las Vegas, NV 89106, USA; baharn1@unlv.nevada.edu (N.B.); badana1@unlv.nevada.edu (A.B.)

**Keywords:** CBCT, malocclusion, adult orthodontics

## Abstract

**Background/Objectives:** This study introduces a novel classification system using cone-beam computed tomography (CBCT) to assess condylar morphology and its correlation with different skeletal classifications. **Methods:** A retrospective CBCT analysis of 288 subjects evaluated condylar shape, flattening at the medial and lateral poles, and the presence of degenerative changes. Statistical analyses identified significant associations. **Results:** Class II skeletal malocclusion was the most prevalent (63.5% females and 36.4% males). Females exhibited a significantly higher prevalence of degenerative changes (*p* < 0.001), with notable lateral pole flattening. The most common condylar morphology was convex (52.43% left and 51% right), followed by angled, round, and flat. Degenerative changes were more frequent on the left side, particularly in Class II Division 1 cases (37%). **Conclusions:** This classification system enhances temporomandibular joint (TMJ) evaluation in orthodontic diagnosis and treatment planning, allowing for the early detection of morphological changes to optimize patient care.

## 1. Introduction

The relationship between the temporomandibular joint (TMJ) and malocclusion is an extremely critical consideration among orthodontic patients with malocclusion. Studies have suggested that the occlusion type may have an impact on the development of temporomandibular disorders (TMD) [1]. TMJ disorder is a common condition that affects the jaw joint and muscles, causing pain and limited jaw movement. It is often associated with malocclusions and other dental problems. Thus, restoring normal occlusal relationships may be a definitive treatment for patients with malocclusion from TMJ abnormalities. No conclusive evidence exists to co-relate TMD with orthodontic treatment [2]. Thus, more longitudinal studies are needed to verify any possible relationship [2]. Investigating condylar morphology in patients with malocclusion—a key element of TMJ—may help examine the relationship between malocclusion and TMJ. There have always been concerns about a correlation between the incidence of TMD problems with malocclusion and orthodontic treatment. A systematic review demonstrated that TMD is neither correlated to orthodontic treatment nor to any specific malocclusion; perhaps the vertical, anteroposterior, and transverse development of the maxilla and mandible can be affected by the different condylar forms regardless of risk for TMD [2].

Panoramic radiography has been widely used as a method to examine the TMJ bony structures. One of the most significant drawbacks of conventional imaging such as panoramic radiography is that it only captures the lateral aspect of the joint [3]. Another major drawback is that it is technique-sensitive. This can be explained by a study where they found that alterations in patient positioning while acquiring panoramic radiographs can simulate the degenerative-change images [4].

Advanced imaging modalities such as CBCT are advantageous as they provide multiplanar imaging. The American Academy of Oral and Maxillofacial Radiology (AAOMR) has advocated CBCT as a “parameter of care” that aids in the diagnosis, treatment planning, and follow-ups for TMJ disorders. Furthermore, CBCT has an excellent spatial resolution and has the capacity to provide diagnostic details in sub-millimeters due to isotropic voxels [5]. CBCT is an imaging technique that can produce images—with high diagnostic quality—of the oral and maxillofacial areas [6]. This imaging technology provides three-dimensional views of the head and neck, including the TMJ and the condylar regions. It can also provide detailed information about the shape and position of the condyles, which can help in the diagnosing and management of malocclusions and other TMJ disorders. CBCT has advantages over traditional medical CT imaging, including lower radiation doses and shorter scanning times, which has made CBCT the gold standard for imaging bone [7]. CBCT is available in many orthodontic practices and is widely used for the assessment of TMJ bone remodeling [8]. Normal condyles are typically rounded and symmetrical, with a smooth contour and a consistent thickness. Abnormalities in condylar shapes—such as flattening, elongation, or deviation—can indicate underlying TMJ disorders and contribute to malocclusion development [9].

CBCT analysis can also reveal other important factors related to the condyles, such as the position of the condyle concerning the skull and the joint space within the TMJ. Two systems have been developed to classify condyle morphology in CBCT imaging. Koyama et al. classified condyle morphology according to the following criteria: N (normal); F (flattening); E (erosion); D (deformity, marginal proliferation, and osteophyte); and S (erosion, deformity, osteophyte, and marginal proliferation) [10]. Ahmad et al. used the following criteria for image analysis of condyle morphology: A (no osteoarthritis); B (indeterminate for osteoarthritis); and C (osteoarthritis) [11]. These classification criteria are used to determine the condylar morphology in patients with malocclusion. The present study aims to investigate the association between condylar shape and various skeletal classifications using CBCT for patients attending the UNLV and SDM clinics and proposes a CBCT condylar-shape classification.

## 2. Materials and Methods

This study was conducted in accordance with the principles of the Declaration of Helsinki. As no additional radiographs or examinations were performed specifically for this study and all collected data were analyzed and presented anonymously, this study was granted exempt status by the Institutional Review Board (IRB) of the University of Nevada, Las Vegas (UNLV), on 4 February 2020 (Protocol No.: 1473778-3).

### 2.1. Study Design and Study Participants

The present cross-sectional study utilized CBCT scans of patients from various age groups who attended the University of Nevada’s Dental Clinic in the Department of Orthodontics. The inclusion criteria for patient selection comprised individuals aged 18 to 40 with a history of malocclusion. The exclusion criteria comprised a history of trauma or condylar fracture, prior maxillofacial surgeries or fractures, diagnosed chronic diseases or congenital disorders affecting bone and cartilage formation, juvenile idiopathic arthritis (JIA) or similar systemic health conditions, and pathological lesions in the condylar and TMJ regions.

### 2.2. Sample Calculation

The sample size was calculated using the *t*-test for repeated measures while considering the following parameters: effect size 0.25, α = 0.05, and power = 0.80. A minimum sample size of 101 individuals was estimated. The sample calculation was performed using G* Power. The sample size was determined by applying the Raosoft formula n *= ^N x^*/_((_*_N_*_− 1)_*_E_*^2^ _+_ *_x_*_)_, taking the margin of error as 5%. For the UNLV datasets, the population size of orthodontic patients with malocclusion meeting the inclusion criteria was 625, and the final sample size for this study was calculated as 244. The actual sample size was 288.

### 2.3. Data Collection and Image Analysis

The data for analysis were extracted from patients’ orthodontic records and included age, gender, ethnicity, and type of malocclusion. De-identification was conducted for all CBCT images. A calibration session was conducted by an experienced board-certified oral maxillofacial radiologist (J.M.) prior to collecting the data. After the calibration session, a methodology and the scoring parameters were established. Two dental students (A.B. and N.B.) independently evaluated the condylar shape, flattening of the condylar heads on the medial and lateral poles, and the presence or absence of degenerative changes. All selected CBCT DICOM datasets were imported into the InVivo5 (version 5.3) (Anatomage Inc., Santa Clara, CA, USA) software, and two calibrated investigators examined all the images independently. The condylar shape was classified into four types—angled, convex, flat, and round—based on the following methodology [12,13,14].

### 2.4. Skeletal Classification

Cephalometric imaging was standardized on the Orthopantomograph OP 300 (Instrumentarium Dental Inc., Milwaukee, WI, USA). The imaging settings were as follows: 65 kV, 2.0 mA, and a 16 s exposure time. As with the CBCT images, imaging was completed in centric occlusion with a cranio-stat holding the patient’s head position aligned to Frankfort horizontal and the sagittal plane parallel to the x-ray film. Steiner published a method of evaluating both hard and soft tissues utilizing cephalometric radiographs [15]. To determine the anteroposterior skeletal classification (Class I, II, and III), Steiner’s analysis uses ANB, which measures the position of the mandible in relation to the maxilla [15].

### 2.5. Proposed CBCT Condylar Shape Classification

**Figure 1:**
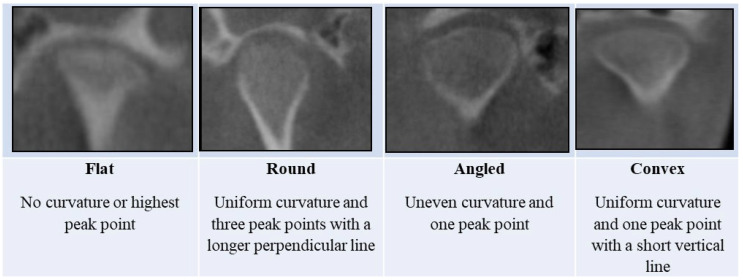
Proposed CBCT classification for the mandibular condylar shape.

The proposed CBCT classification for the mandibular condylar shape is shown in Figure 1—this includes the following condyle, with no curvature or highest peak, which was considered a flat condyle; three peak points with a longer perpendicular line on a uniform curvature was classified as a rounded condyle; a condyle with uneven curvature and one peak point was classified as an angled condyle; and a convex condyle was classified as when the condyle had uniform curvature and one peak point with a short vertical line.

### 2.6. Acquisition

The iCAT Next Generation (Imaging Sciences International, Hatfield, PA, USA) CBCT unit was used to capture all scans. The iCAT standardized protocol included the extended (17 × 23 cm) field of view with a 0.3-mm slice thickness and a 26.9-s acquisition time. The third-party CBCT reconstruction software InVivo5.0 (version 5.3) (Anatomage, San Jose, CA, USA) was used to evaluate the DICOM-3 format of the all-saved scans.

### 2.7. CBCT Scan Evaluation

To analyze the scans, first, the scan was presented into the reconstruction program in the sagittal view keeping the horizontal reference line passing through the middle portion of the palate. Next, the standardized layout of frontal, axial, and 6 laterals was selected on the TMJ view. Later, adjustments were made to the TMJ view of the software where the area of the TMJ was placed in the view. While analyzing the data, the slice section was taken from the middle of the condyle on the axial view. Then, the condylar head shape was determined by looking at the slice view using the parameters of width = 0.8 mm, interval = 0.25 mm, and thickness = 0 mm [16]. The CBCT scan was then evaluated by viewing the condyle in the coronal plane and categorizing it into one of four shapes: flat, round, angled, or convex [16]. The flattening and degenerative changes were analyzed on the sagittal view by viewing the slices of the medial and lateral poles. Any changes in the condylar head except flattening were taken as degenerative changes, including subchondral sclerosis, subchondral cysts, erosions, and osteophytes, even if one radiographic feature was present.

### 2.8. Statistical Analysis

Data from the 288 participants’ images were used for descriptive and inferential statistical analyses. SPSS Statistics version 20.0 (IBM Corp., Armonk, NY, USA) was used for the data analysis, applying a chi-square test, and Microsoft Word and Excel software was used to generate graphs and tables.

## 3. Results

Among the 288 samples, the most prominent class according to the skeletal classification was Class II (70.1%) (Table 1). The distribution of condylar shapes in relation to the skeletal classification is shown in Table 1.

Irrespective of the skeletal classification, the most common condylar shape was convex (52.43% on the left condyle and 51% on the right condyle), followed by angled (27.43% on the left and 32.3% on the right condyle), round (13.89% on the left and 12.5% on the right condyle), and flat (6.25% on the left and 4.2% on the right condyle). These results were statistically significant with *p* values less than 0.0001. This study also evaluated the flattening of the medial and lateral poles in Class I, II, and III patients. The absence of flattening of the medial pole of the condylar head in Class I, II, and III patients was statistically significant on both sides (right and left); however, lateral pole flattening was present in these patients.

**Figure 2:**
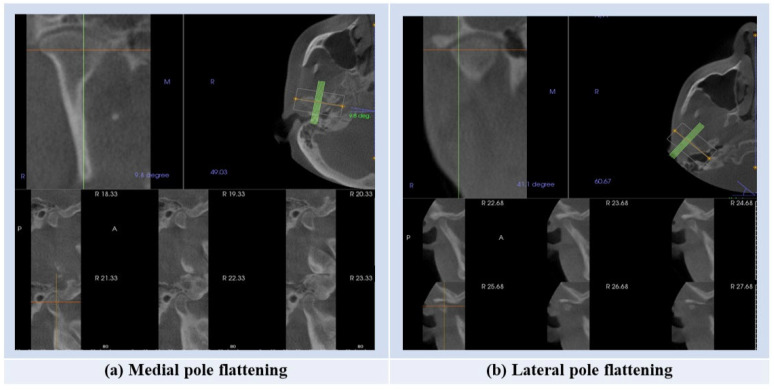
The steps for assessment of the presence of the condylar flattening.

The flattening of the medial pole was present in the left condyle in 33.3% of cases, while for the right condyle, flattening of the medial pole was present in 39.3% of cases (Table 1, Figure 2).

**Figure 3:**
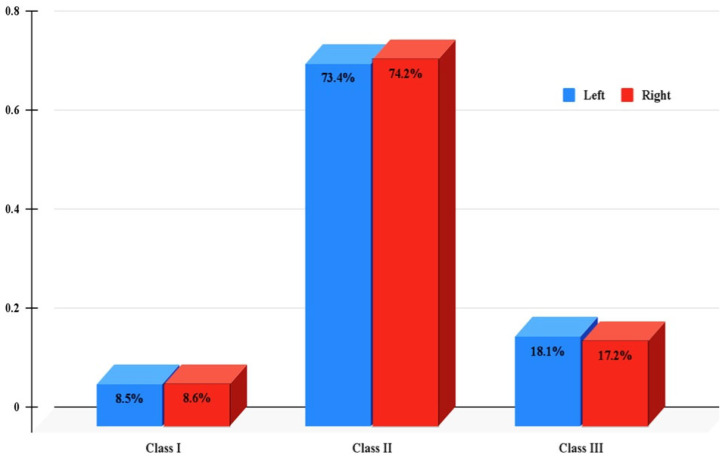
The presence of condylar degenerative changes in relation to skeletal classification.

Figure 3 shows that Class II had the highest degenerative changes followed by Class III and Class I, respectively.

The chi-square test was used to evaluate and compare the different measuring parameters (Table 2). For both left and right condyles, the convex shape was the most common shape, followed by the angled shape, and the difference was statistically significant (Table 2).

The flattening of the medial and lateral poles appeared in 33.3% and 47.9% of the left condyle, respectively. In contrast, for the right condyle, the flattening of the medial and lateral poles appeared in 39.2% and 46.5%, respectively (Table 3). Degenerative changes for both the left and right condyles were present in 32.6% and 32.3%, respectively (Table 3).

The chi-square test examined the linear association of the degenerative changes in both condyles with different demographic parameters. Females had a high percentage of degenerative changes compared to males, and the differences were statistically significant for the right condyle (Table 3). There was a statistical difference in the presence of degenerative changes among different age groups for both left and right condyles (Table 3).

Table 3 shows the distribution of Class II malocclusion cases into the 1, 2, and open-bite subdivisions. A total of 59% of Class II malocclusion cases were classified as Division 1, followed by Division 2 (33.6%) and open-bite (7.4%). More degenerative changes were noticed on the left side of Division 1 (37%), followed by the Division 2 and open-bite subdivisions (Table 3).

## 4. Discussion

The shape of the condyle was described on the basis of the superior surface of the condylar head and was categorized into four shapes, namely angled, convex, flat, and round [12]. In the present investigation, the most common condylar shape was convex, which agrees with other studies that confirmed that among the condylar morphologies, a convex shape is the most common shape [12,16,17]. There are several factors that may affect the shape of the condyle over time, and these morphological changes lead to remodeling of the bone. The variability depends on the age, facial type, gender, malocclusion type, occlusal force, and functional load [18,19]. It has also been stated that parafunctional habits may lead to condylar and articular eminence degenerative changes [20]. These degenerative changes, which are also known as osteoarthritis, are the most common pathology affecting the TMJ [18,21]. For the present investigation, the presence or absence of osteoarthritis was evaluated following the criteria described by Wiese et al. [22]. For the accurate diagnosis and appropriate treatment planning of any disease-related dysfunction, it is important to identify in-depth any pathologies or bone changes.

The effects of female hormones, especially estrogen, in mediating TMJ degeneration have been questionable in the literature [23,24]. The present investigation showed that degenerative changes were more predominant in females and the result was statistically significant for the right side of the condylar head. These results are in accordance with various other conducted studies [22,25,26,27]. The dental status, patient status, and number of teeth present all contribute to the changes in condylar contour due to the functional demands. Such alterations help to redistribute the load to the TMJ joints. The degenerative changes were more common with Class II malocclusions, followed by Class I and Class III, respectively. The present results are in accordance with a scoping review that concluded that skeletal Class II malocclusions can be risk factors for developing radiographically detectable degenerative changes [28].

In the present research, the degenerative changes were more associated with Class II Division 1 malocclusion cases, where the left side of the condylar head was more affected. Previous studies have indicated that longer exposure to malocclusion is associated with more extensive changes in the TMJ [29,30]. A limitation of our study was that all participants were orthodontic patients. Future research should include patients with long-term malocclusion to provide a more comprehensive understanding. Additionally, future studies should investigate the relationships between vertical growth patterns, sagittal class relationships, and TMJ morphology. Several studies have concluded that orthodontic treatment over a longer span of time has the ability to lead to changes in the morphology of the condylar head [28,31]. Within the scope of the present investigation, the proposed CBCT condylar shape classification should be utilized for TMJ analysis before, during, and after orthodontic treatment.

## 5. Conclusions

The current research highlights the importance of TMJ evaluation and condylar shape classification in the pre- during, and post-orthodontic treatment phases. This evaluation can help to assess any morphological changes in the condylar head in response to specific orthodontic treatment.

## Figures and Tables

**Table 1:** Distribution of condylar shape configurations and presence of a condylar flattening pole in relation to skeletal classification.

	Class I		Class II		Class III		Total (%)	
	Left (%)	Right (%)	Left (%)	Right (%)	Left (%)	Right (%)	Left (%)	Right (%)
Condylar Shape Configuration								
Angled	4 (5.1%)	5 (5.4%)	52 (65.8%)	65 (69.9%)	23 (29.1%)	23 (24.7%)	79 (27.4%)	93 (32.3%)
Convex	15 (9.9%)	16 (10.9%)	104 (68.9%)	100 (68%)	32 (21.2%)	31 (21.1%)	151 (52.4%)	147 (51%)
Flat	2 (11.1%)	1 (8.3%)	12 (66.7%)	9 (75%)	4 (22.2%)	2 (16.7%)	18 (6.3%)	12 (4.2%)
Round	3 (7.5%)	2 (5.6%)	34 (85%)	28 (77.8%)	3 (7.5%)	6 (16.7%)	40 (13.9%)	36 (12.5%)
Total	24 (8.3%)	24 (8.3%)	202 (70.1%)	202 (70.1%)	62 (21.5%)	62 (21.5%)	288 (100%)	288 (100%)
Flattening Pole								
Medial	8 (8.3%)	8 (7.1%)	65 (67.7%)	81 (71.7%)	23 (24%)	24 (21.1%)	96 (100%)	113 (100%)
Lateral	9 (6.5%)	9 (6.7%)	99 (71.7%)	95 (70.9%)	30 (21.7%)	30 (22.4%)	138 (100%)	134 (100%)

**Table 2:** Chi-square test evaluating different measuring parameters.

		N (%)	x^2^ Value	*p* Value
Condyle shape–left	angled	79 (27.4%)	142.08	<0.0001 **
	convex	151 (52.4%)		
	flat	18 (6.3%)		
	round	40 (13.9%)		
Condyle shape–right	angled	93 (32.3%)	152.25	<0.0001 **
	convex	147 (51%)		
	flat	12 (4.2%)		
	round	36 (12.5%)		
Flattening of left medial pole	n	192 (66.7%)	32.00	<0.0001 **
	p	96 (33.3%)		
Flattening of left lateral pole	n	150 (52.1%)	0.50	0.480
	p	138 (47.9%)		
Flattening of right medial pole	n	175 (60.8%)	13.35	<0.001 **
	p	113 (39.2%)		
Flattening of right lateral pole	n	154 (53.5%)	1.39	0.239
	p	134 (46.5%)		
Degenerative changes–left condyle	n	194 (67.4%)	34.72	<0.0001 **
	p	94 (32.6%)		
Degenerative changes–right condyle	n	195 (67.7%)	36.12	<0.0001 **
	p	93 (32.3%)		

Note: (n = not present; p = present). Chi-square test—*p* > 0.05, not significant; ** *p* < 0.001, highly significant).

**Table 3:** Linear associations between different demographics among the examined cases using chi-square test.

		Degenerative Changes–Left Condyle			Degenerative Changes–Right Condyle		
		Not Present (%)	Present (%)	*p* Value	Not Present (%)	Present (%)	*p* Value
Sex	Female	121 (66.1%)	62 (33.9%)	0.553	116 (63.4%)	67 (36.6%)	0.038 *
	Male	73 (69.5%)	32 (30.5%)		79 (75.2%)	26 (24.8%)	
Age	≤20	79 (72.5%)	30 (27.55)	0.012 *	83 (76.1%)	26 (23.9%)	0.009 *
	21–30	68 (73.1%)	25 (26.9%)		63 (67.7%)	30 (32.3%)	
	31–40	25 (25.8%)	19 (43.2%)		27 (61.4%)	17 (38.6%)	
	41–50	14 (50%)	14 (50%)		14 (50%)	14 (50%)	
	51–60	5 (50%)	5 (50%)		4 (40%)	6 (60%)	
	>60	3 (75%)	1 (25%)		4 (100%)	0 (0%)	
Skeletal AP classification	Class I	16 (66.5%)	8 (33.3%)	0.421	16 (66.5%)	8 (33.3%)	0.311
	Class II	133 (65.8%)	69 (34.5%)		133 (65.8%)	69 (34.5%)	
	Class III	45 (72.6%)	17 (27.4%)		46 (74.2%)	16 (25.8%)	
Ethnicity	African American	20 (64.5%)	11 (35.5%)	0.970	21 (67.7%)	10 (32.3%)	0.298
	Asian	14 (66.7%)	7 (33.3%)		16 (76.2%)	5 (23.8%)	
	Caucasian	60 (66.7%)	30 (33.3%)		61 (67.8%)	29 (32.2%)	
	Hispanic	99 (68.8%)	45 (31.3%)		97 (67.4%)	47 (32.6%)	
	Native Indian	1 (50%)	1 (50%)		0 (0%)	2 (100%)	

Note: *p* > 0.05, not significant; * *p* < 0.05, significant.

## Data Availability

All data have been included in this study.

## Abbreviations

The following abbreviations are used in this manuscript:

CBCT	Cone-beam computed tomography
TMD	Temporomandibular disorders
TMJ	Temporomandibular joint
DICOM	Digital Imaging and Communications in Medicine
